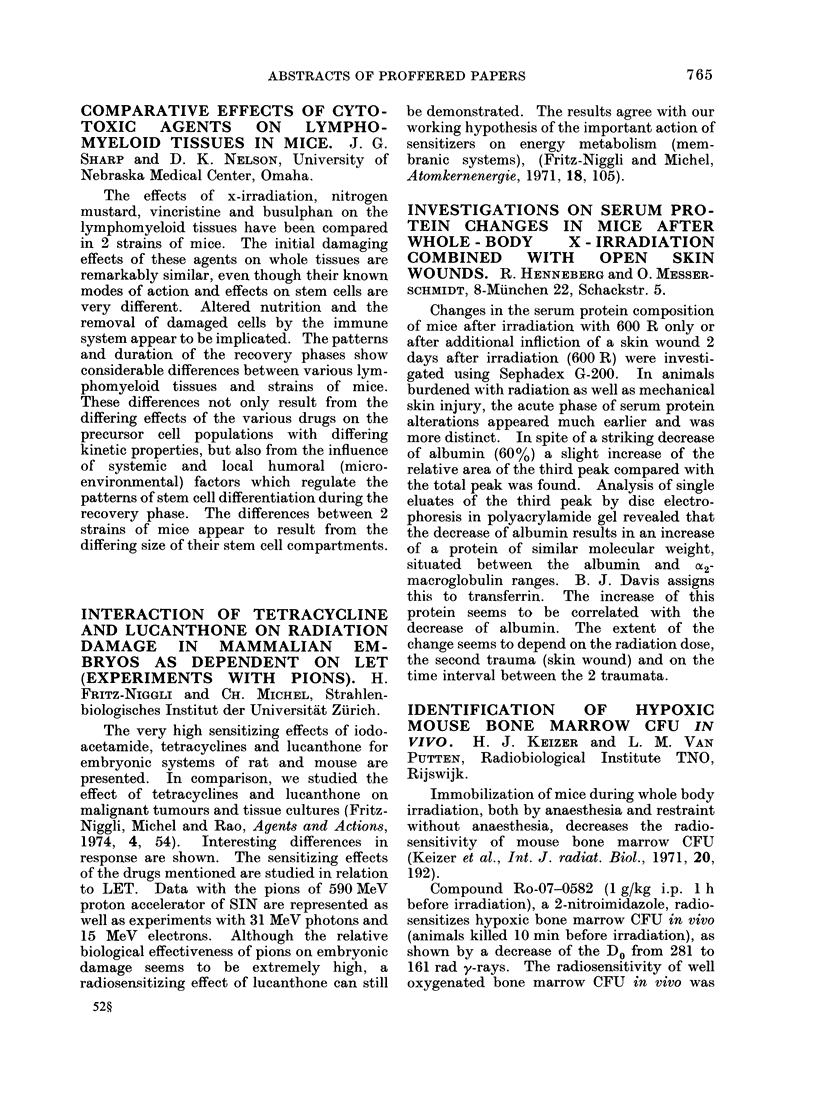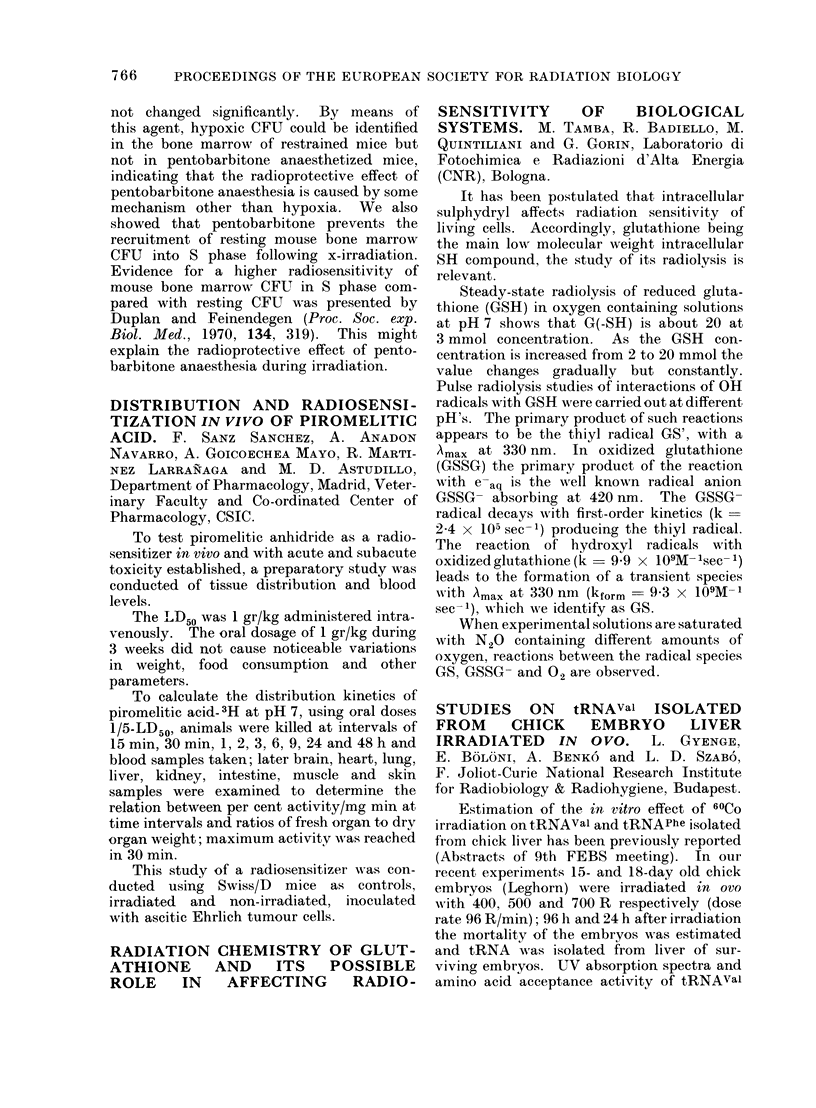# Proceedings: Identification of hypoxic mouse bone marrow CFU in vivo.

**DOI:** 10.1038/bjc.1975.337

**Published:** 1975-12

**Authors:** H. J. Keizer, L. M. Van Putten


**IDENTIFICATION OF HYPOXIC MOUSE BONE MARROW CFU *IN VIVO*.** H. J. Keizer and L. M. Van Putten, Radiobiological Institute TNO, Rijswijk.

Immobilization of mice during whole body irradiation, both by anaesthesia and restraint without anaesthesia, decreases the radiosensitivity of mouse bone marrow CFU (Keizer *et al.*, *Int. J. radiat. Biol.*, 1971, **20**, 192).

Compound Ro-07–0582 (1 g/kg i.p. 1 h before irradiation), a 2-nitroimidazole, radiosensitizes hypoxic bone marrow CFU *in vivo* (animals killed 10 min before irradiation), as shown by a decrease of the $D_0$ from 281 to 161 rad $\gamma$-rays. The radiosensitivity of well oxygenated bone marrow CFU *in vivo* was not changed significantly. By means of this agent, hypoxic CFU could be identified in the bone marrow of restrained mice but not in pentobarbitone anaesthetized mice, indicating that the radioprotective effect of pentobarbitone anaesthesia is caused by some mechanism other than hypoxia. We also showed that pentobarbitone prevents the recruitment of resting mouse bone marrow CFU into S phase following x-irradiation. Evidence for a higher radiosensitivity of mouse bone marrow CFU in S phase compared with resting CFU was presented by Duplan and Feinendegen (*Proc. Soc. exp. Biol. Med.*, 1970, **134**, 319). This might explain the radioprotective effect of pentobarbitone anaesthesia during irradiation.